# Editorial: Development of novel small molecules as therapeutics for inflammatory diseases and delineating their molecular mechanisms

**DOI:** 10.3389/fphar.2022.1088908

**Published:** 2022-11-22

**Authors:** Tharmarajan Ramprasath, Nandakumar Natarajan, Jacob Gopas, Ramasamy Subbiah, Peramaiyan Rajendran, Sakthivel Vaiyapuri

**Affiliations:** ^1^ Center for Molecular and Translational Medicine, Georgia State University, Atlanta, GA, United States; ^2^ The University of Texas Health Science Center at Tyler, Tyler, TX, United States; ^3^ Ben-Gurion University of the Negev, Beersheba, Israel; ^4^ Department of Biochemistry, School of Biological Sciences, Madurai Kamaraj University Madurai, Madurai, India; ^5^ Department of Biological Sciences, College of Science, King Faisal University, Al Ahsa, Saudi Arabia; ^6^ University of Reading, Reading, United Kingdom

**Keywords:** NF-κB, inflammation, small molecules, NLRP3, vascular diseases

## Introduction

Inflammation is a critical and rapid physiological response of our immune system to any infection or tissue injury which is associated with various diseases (Zhu and Hou, 2021). However, when the inflammatory response fails to resolve the cause of inflammation, it can be detrimental and lead to the development of many diseases (Hanke et al., 2016; Ramprasath et al., 2021). Though, currently many approved anti-inflammatory drugs are in clinical use (e.g., non-steroidal anti-inflammatory drugs; NSAIDs), (Kohler et al., 2016), the long-term use of anti-inflammatory drugs are associated with many side effects including, gastrointestinal reactions and damages to the cardiovascular system (Wongrakpanich et al., 2018). In medicinal chemistry, the discovery based on the small molecule approach has opened the door to a new way to develop novel therapeutics for people with severe inflammatory conditions. In recognition of the clinical value of this study, this subject featured fourteen original research and two review articles in the field of inflammation and small molecule study with a larger perspective to generate ideas for future scientific and clinical research.

### Small molecule inhibitors targeting the NLRP3 inflammasome

NF-κB is a key activator of inflammation that primes the NLR pyrin domain 3 (NLRP3)-inflammasome for its activation (Zhong et al., 2016). In this special issue, different research groups published their work focused mainly on inflammasome. Zhao et al. investigated a compound named FxUD, which suppressed the NF-κB/NLRP3 signaling pathway and lowered the serum uric acid level to alleviate renal inflammation in hyperuricemic mice. Similarly, another compound KPT-8602, was validated for its potential to inhibit the activation of the NF-κB/NLRP3 signaling. The administration of KPT-8602 attenuated both lipopolysaccharide (LPS)-induced peripheral inflammation and 1-methyl-4-phenyl-25 1,2,3,6-tetrahydropyridine (MPTP)-induced neuroinflammation *in vivo* (Liu et al *.*). Another study tested a compound called MCL against radiation-induced enteropathy (RIE), one of the fatal complications of treatment for abdominal and pelvic tumors. In the mouse RIE model, MCL-mediated autophagy ameliorated RIE by NLRP3 inflammasome degradation (Wu et al *.*). An inflammasome inhibitor 3,4-methylenedioxy-β-nitrostyrene (MNS) was shown to be more efficient in inhibiting the secretion of interleukin-1β (IL-1β) by blocking oligomerization of apoptosis-associated speck-like protein (ASC) than other inhibitors NLRP3-IN-2 and JC124. In this study, Zheng et al *.* demonstrated using the dextran sulfate sodium (DSS)-induced colitis model that MNS alleviated DSS-induced intestinal inflammation by inhibiting NLRP3 inflammasome activation, which may function as an effective therapeutic for IBD. Lin et al *.* study demonstrated the efficacy of compound 149-01 in alleviating LPS-induced systemic inflammation, monosodium urate crystals (MSU)-induced peritonitis and experimental autoimmune encephalomyelitis (EAE) by preventing the interactions between NLRP3 and NEK7.

### Small molecule modulators for COVID-19

Trichomicin, a novel small-molecule compound, is isolated from the fungus *Trichoderma harzianum* (Zhu et al., 2020). Trichomicin was explored for its inhibitory effect on cytokine expression by Chen et al. This compound could inhibit the Stat3 and NF-κB phosphorylation and showed the potential to treat the patients with cytokine release syndrome, a significant cause of COVID-19 disease severity. Vascular inflammation is one of the unusual symptoms among COVID-19 survivors. Ragavan et al. showed that histamine and histamine receptor signaling is likely to be essential for SARS-CoV-2 spike protein S1 Receptor-Binding Domain (Spike) protein to induce ACE2 internalization in endothelial cells, which causes endothelial dysfunction. This effect was blocked by treating with famotidine (a histamine H2 receptor blocker), an antiviral drug (Mukherjee et al., 2021), in cultured human coronary artery endothelial cells.

### Small molecule inhibitors for inflammatory diseases

ACT-1004-1239 is a compound that showed a therapeutic effect against LPS inhalation-induced lung vascular injury *in vivo*. The authors provided data to show that this compound can alleviate acute lung injury (ALI) and its more severe form, acute respiratory distress syndrome (ARDS) (Pouzol et al *.*). Similarly, another compound, M20 interact with the MyD88-Toll/interleukin-1 receptor domain and thereby inhibits the protein dimerization, which could serve as a potential strategy for the treatment of acute lung injury (Song et al *.*).

Zhou et al *.* demonstrated that *topical* application of an anti-inflammatory compound isolated from the Chinese herb *Sophora alopecuroides L.* suppressed epidermal proliferation, erythema and infiltration of inflammatory cells in skin lesions, thereby alleviating imiquimod (IMQ)-induced psoriasis in mice. Huang et al *.* showed the implication of a plant-derived compound, N-acetyldopamine dimer (NADD from *Isaria cicadae)*, for its inhibitory effects on the expression of genes in inflammation-related signaling under *in vivo* settings for ulcerative colitis.

Saikosaponin D (SSD), an active ingredient isolated from Bupleurum, could activate the Nrf2/HO-1/ROS axis, further inhibiting the production of inflammatory mediators and protecting against ECM destruction. SSD delayed the progression of osteoarthritis (OA) in DMMs model mice *in vivo*. Therefore, the authors claim SSD could have potential for the treatment of OA (Wu et al.). A Study by Lilley et al.demonstrated the role of orphan nuclear receptor 4A2 (NR4A2/Nurr1) in an animal model of RA. The study validated the hTNF-a animal model for testing small molecules and genetic strategies for targeting NR4A2*.* Zhao et al *.* study highlighted the α2M-rich serum (α2MRS) autologous joint injection to treat post-traumatic osteoarthritis. This article provided a basis for the clinical translation of α2MRS against cartilage degeneration.

Jayusman et al. provided a comprehensive review of the studies related to effects of different polyphenolic substances on periodontal inflammation. The review also explored the pharmaceutical significance of polyphenol-loaded nanoparticles in controlling periodontitis. Focusing on the effect of Dexmedetomidine (DEX) on Immune cells, Chen et al *.* comprehensively reviewed the published human and animal studies related to DEX, and its role in related diseases, and discussed the potential research direction.

## Conclusion and future perspective

Small molecules offer numerous advantages compared to currently used biological therapies. Identifying small molecules with shorter half-lives helps the patients stop taking their medication quickly, which could offer better medical management. Hence, employing advanced and novel approaches in the development of small molecule therapeutics is urgently needed. We hope that the scientific knowledge provided in this special issue will encourage many researchers to address the several outstanding challenges in this field to advance scientific research on inflammatory signaling events and small molecule interactions to promote better therapeutic development.
